# Novel condylar repositioning method for 3D-printed models

**DOI:** 10.1186/s40902-018-0143-7

**Published:** 2018-03-05

**Authors:** Keisuke Sugahara, Yoshiharu Katsumi, Masahide Koyachi, Yu Koyama, Satoru Matsunaga, Kento Odaka, Shinichi Abe, Masayuki Takano, Akira Katakura

**Affiliations:** 1 Department of Oral Pathobiological Science and Surgery, Tokyo Dental College, 2-9-18 Kanda Misaki-cho, Chiyoda-ku, Tokyo, Japan; 2 Oral Health Science Center, Tokyo Dental College, 2-9-18 Kanda Misaki-cho, Chiyoda-ku, Tokyo, Japan; 3 Department of Anatomy, Tokyo Dental College, 2-9-18 Kanda Misaki-cho, Chiyoda-ku, Tokyo, Japan; 4 Department of Oral and Maxillofacial Surgery, Tokyo Dental College, 2-9-18 Kanda Misaki-cho, Chiyoda-ku, Tokyo, Japan

**Keywords:** Three-dimensional models, Condylar repositioning, Tumor resection, Orthognathic surgery

## Abstract

**Background:**

Along with the advances in technology of three-dimensional (3D) printer, it became a possible to make more precise patient-specific 3D model in the various fields including oral and maxillofacial surgery. When creating 3D models of the mandible and maxilla, it is easier to make a single unit with a fused temporomandibular joint, though this results in poor operability of the model. However, while models created with a separate mandible and maxilla have operability, it can be difficult to fully restore the position of the condylar after simulation. The purpose of this study is to introduce and asses the novel condylar repositioning method in 3D model preoperational simulation.

**Methods:**

Our novel condylar repositioning method is simple to apply two irregularities in 3D models. Three oral surgeons measured and evaluated one linear distance and two angles in 3D models.

**Results:**

This study included two patients who underwent sagittal split ramus osteotomy (SSRO) and two benign tumor patients who underwent segmental mandibulectomy and immediate reconstruction. For each SSRO case, the mandibular condyles were designed to be convex and the glenoid cavities were designed to be concave. For the benign tumor cases, the margins on the resection side, including the joint portions, were designed to be convex, and the resection margin was designed to be concave. The distance from the mandibular ramus to the tip of the maxillary canine, the angle created by joining the inferior edge of the orbit to the tip of the maxillary canine and the ramus, the angle created by the lines from the base of the mentum to the endpoint of the condyle, and the angle between the most lateral point of the condyle and the most medial point of the condyle were measured before and after simulations. Near-complete matches were observed for all items measured before and after model simulations of surgery in all jaw deformity and reconstruction cases.

**Conclusions:**

We demonstrated that 3D models manufactured using our method can be applied to simulations and fully restore the position of the condyle without the need for special devices.

## Background

In the field of oral and maxillofacial surgery, many institutions have recently begun using three-dimensional (3D) printers to create 3D models of a variety of diseases. The first medical fabrication laboratory in Japan was established at Tokyo Dental College—the “Fab Lab TDC”—in December 2013 [1]. Techniques to construct full-scale 3D models, such as of the jaw, based on computed tomography (CT) and magnetic resonance imaging (MRI) modalities have been reported recently [1–3]. We have also created preoperative 3D-printed models of cases for tumors in maxilla and mandible, jaw deformities, and used them primarily consultation to patients and for preoperative simulations.

When creating 3D models of the mandible and maxilla, it is easier to make a single unit with a fused temporomandibular joint, though this results in poor operability of the model and when bending reconstructive plates. However, while models created with a separate mandible and maxilla have good operability, it can be difficult to fully restore the position of the mandibular condyle after simulations. To fully restore the position of the condyle, methods have been reported that include the manufacture of a device to maintain the configuration of the mandible and embedding of magnets into the joint [3].

Here, we report a novel method we developed to fully restore the position of the condyle that does not require any device other than the 3D model. We also examined the reproducibility of the method.

## Methods

### Subjects

This study included two patients who underwent sagittal split ramus osteotomy (SSRO) and two benign mandibular tumor patients who underwent segmental mandibulectomy and immediate reconstruction. The study was approved by the ethics committee of Tokyo Dental College (No. 646) (Tokyo, Japan).

### Methods

**Fig. 1:**
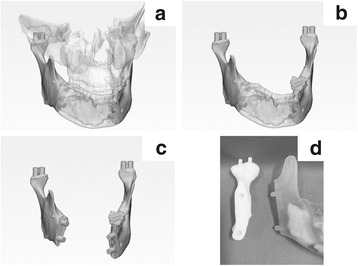
**a**–**c** The margins on the resection side, including the joint portions, were designed to be convex, and the resection margin was designed to be concave using 3-matic®. **d** 3D models were created using OBJET Connex 260®

Image data of the maxilla, mandible, and lesions were reconstructed from CT image data using Mimics® (Materialise, Leuven, Belgium). Our novel condylar repositioning method is simple to apply two irregularities in 3D models. For each SSRO case, the mandibular condyles were designed to be convex and the glenoid cavities were designed to be concave using 3-Matic® (Materialise, Leuven, Belgium) (Fig. 1a–c). For the mandibular tumor cases, the margins on the resection side, including the joint portions, were designed to be convex, and the resection margin was designed to be concave using 3-matic®. 3D models were created using OBJET Connex 260® (Staratasys, MI) (Fig. 1d).

### Measurements

**Fig. 2:**
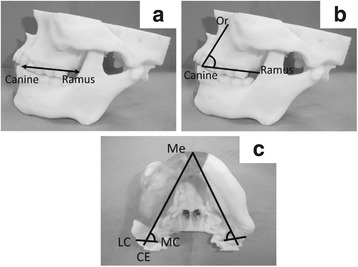
**a**–**c** Measurements, length, and angle. **a** Length, ramus to maxillary canine, **b** Angle, Or-Canine-Ramus, **c** Angle, Me-CE and LC-MC

The distance from the mandibular ramus to the tip of the maxillary canine, the angle created by joining the inferior edge of the orbit (Or) to the tip of the maxillary canine and the ramus, the angle created by the lines from the base of the mentum (Me) to the endpoint of the condyle (CE), and the angle between the most lateral point of the condyle (LC) and the most medial point of the condyle (MC) [4] were measured before and after simulations by three certified oral surgeons (Fig. 2a–c).

## Results

### Evaluation of the length and angle (Table 1)

For all four cases, the mean values of each item measured by the three oral surgeons were calculated before and after model operations. Only the affected side of the reconstruction cases was measured.

**Table 1:** Measurement results

				Length, ramus to maxillary canine				Angle, Or-Canine-Ramus				Angle, Me-CE and LC-MC			
				Before simulation		After simulation		Before simulation		After simulation		Before simulation		After simulation	
Case	Diagnosis	Age	Gender	R (mm)	L (mm)	R (mm)	L (mm)	R (°)	L (°)	R (°)	L (°)	R (°)	L (°)	R (°)	L (°)
1	Mandibular ameloblastoma	29	Male	–	53.5	–	53.5	–	68	–	68	78	86	78	86
2	Mandibular myxoma	49	Male	–	53	–	53	–	64.5	–	64.5	68	71	68	71
3	Jaw deformity (mandibular prognathism)	23	Female	46	48	46	48	71	73	71	72.5	69	71	69	71
4	Jaw deformity (mandibular prognathism and asymmetry)	25	Female	47	43	47	43	64	63	64	63	75.5	76	75.5	76

#### Length of the ramus to the maxillary canine

The distances from the anterior edge of the ramus to the canine before and after model simulations of surgery matched completely in all four cases.

#### Canine-orbitale and ramus-canine angles

The canine-orbitale and ramus-canine angles before and after simulations matched completely in three cases. A decrease of 0.5° was observed in case 3 (mandibular tumor patient).

#### Me-CE and LC-MC angles

The Me-CE and LC-MC angles before and after model simulations of surgery matched completely in all four cases.

Near-complete matches were observed for all items measured before and after model simulations for surgery in all jaw deformity and reconstruction cases. These results indicate that this method can solve the problems associated with previous methods, which required devices other than a 3D model and relied on uncertain positioning of the condyle based on the operator’s visual estimates.

## Discussion

Globally, customized metal plates for jaw deformation and reconstruction are made with CAD/CAM based on preoperative computer simulations [5–7]. However, in Japan, only premade reconstructive plates can be used in patients. Most of the commercially available system is usefulness for operator who has a certain level of technique, but it is difficult for inexperienced operator to add image anatomical structure. Actually, young operator simulates for the operation with 3D model, to easily and deeply understand and operate safely. Use of full-scale 3D models facilitates not only a shorter, more precise operation as a result of a realistic pre-operation simulation, but enables a better grasp of the extent of bone movement and illustrates more ideal occlusion. 3D models would also enable the construction of temporary crowns and the fabrication of operation plates before surgery. In other institution, they used a magnet for condylar placement. This method is easier than our method. However, our method has higher accuracy and is cheaper than a magnet method.

Anatomically, the position of the mandible with respect to the maxilla is maintained only by ligaments. If the proximal bone fragment that includes the ramus detaches, the force of dislocation or rotation may cause postoperative jaw deformity or progressive condylar resorption [8]. Therefore, errors in preoperative simulations can affect the patient’s postoperative oral function.

In the present study, we demonstrated that 3D models manufactured using our method can be applied to simulations and fully restore the position of the condyle without the need for special devices. The bending of metal plates can also be performed in this state. We plan to apply this method to many patients and examine the surgical precision using superimposition on postoperative CT.

## Conclusions

3D model should have been used with this technique of condyle repositioning method for simulation of reconstruction and orthognathic surgery case. The novel repositioning method appears to be a simple and efficient way to correct condylar position.

## Abbreviations

3D: Three-dimensional
CAD/CAM: Computer-aided design/computer-aided manufacturing
CE: Endpoint of the condyle
CT: Computed tomography
LC: Lateral point on the condyle
Me: Base of the mentum
Or: Inferior edge of the orbit
SSRO: Sagittal split ramus osteotomy
